# Accuracy of Gingival Crevicular Fluid Biomarkers of MMP8, TIMP1, RANK, RANKL, and OPG in Differentiating Symptomatic and Asymptomatic Apical Periodontitis

**DOI:** 10.3390/diagnostics14171872

**Published:** 2024-08-27

**Authors:** Zeena Tariq Abdulhadi, Anas Falah Mahdee, Sarhang Sarwat Gul

**Affiliations:** 1 Department of Restorative and Aesthetic Dentistry, College of Dentistry, University of Baghdad, Baghdad 10071, Iraq; zeenaalani@yahoo.com (Z.T.A.) a.f.mahde@codental.uobaghdad.edu.iq (A.F.M.); 2 Medical Laboratory Department, College of Health and Medical Technology, Sulaimani Polytechnic University, Sulaymaniyah 46001, Iraq; 3 Department of Periodontics, College of Dentistry, University of Sulaimani, Sulaymaniyah 46001, Iraq

**Keywords:** accuracy, endodontic, GCF, apical periodontitis, biomarkers

## Abstract

Apical periodontitis (AP) is the most prevalent chronic inflammatory disease of the teeth. Bone resorption dynamics in symptomatic and asymptomatic AP are still unrecognized. This study examined different inflammatory markers within gingival crevicular fluid, including matrix metalloproteinases 8 (MMP8), tissue inhibitors of metalloproteinases 1 (TIMP1), receptor activator of nuclear factor κB (RANK), its ligand (RANKL), and osteoprotegerin (OPG), to be used in comparing symptomatic apical periodontitis (SAP) and asymptomatic apical periodontitis (AAP) versus healthy teeth. Subjects with SAP, AAP, and a control group were recruited and GCF samples were collected by Periopaper strips. Clinical and radiographical measures were used for diagnosing AP. Levels of MMP8, TIMP, RANK, RANKL, and OPG were determined by ELISA and their abilities to discriminate between examined sites were evaluated by receiver operator characteristic (ROC) curves. All examined biomarkers were statistically significant higher (*p* < 0.05) in SAP than AAP and the control group, apart from RANK. Significant positive correlations (*p* < 0.05) were identified between all SAP and AAP biomarkers except TIMP1 and RANK in AAP teeth. TIMP1 and OPG exhibited the highest ability to distinguish between SAP and AAP with areas under the curve of 0.824 and 0.763 in comparing SAP and the control group, and 0.732 and 0.73 when comparing AAP and the control group, respectively. Additionally, TIMP1 and OPG showed the highest AUC of 0.778 and 0.747 when SAP and AAP were compared, respectively. This study concluded that GCF levels of TIMP1 and OPG can be used to differentiate between SAP, AAP, and healthy teeth.

## 1. Introduction

Apical periodontitis (AP) is the most prevalent dental disease caused by a microbial infection of the dental pulp, characterized by periodontal inflammation in the apical region of the affected tooth, whereas the other less frequent causes of AP could be trauma or a perio-endo lesion [1,2,3]. Since the bacteria cannot be completely eradicated, the host attempts to contain the infection and avoid it from growing, even at the cost of apical tissue destruction and the development of osteolytic AP, which is believed to be the distinguishing feature of persistent forms of apical periodontitis [4,5,6].

From a clinical perspective, AP can be classified as either asymptomatic apical periodontitis (AAP) or symptomatic apical periodontitis (SAP), depending upon whether or not the disease relates to clinical symptoms [6]. It has been reported that this clinical variability could be related to the bacterial consortia and the host’s response interaction [7]. Studies support that symptomatic apical periodontitis is associated with changes in the bacterial load and diversity and the host’s immune response [6,7,8]. This response is mediated by numerous cell types that produce a milieu of proinflammatory cytokines, chemokines, and neuropeptides [9]. However, the current diagnostic method for these conditions depends solely on the patient’s symptoms rather than more realistic inflammatory screening investigations. There are several cases that could be treated in a single visit, while others could develop post-treatment complications [10]. Therefore, if the operator has more diagnostic tools that could reflect the actual inflammatory process in the apical region, their treatment decision will be more realistic, especially in cases with unclear symptoms.

Gingival crevicular fluid (GCF) is an important natural fluid that can display the event mechanism in situ via the investigation of its content and conformation accompanied by the flow rate [11,12]. Upregulated cytokine expression shifts the periodontal turnover rate, eventually leading to the degradation of extracellular matrix components by secreted proteases such as matrix metalloproteinases (MMPs) [6,9]. A special emphasis has been placed on the involvement of collagenases, particularly MMP-8 and MMP-13, and gelatinases MMP-2 and MMP-9, in periodontal diseases since type I collagen comprises the vast majority of the extracellular matrix in periodontal tissues. One of the most promising biomarkers for periodontitis in oral fluids has been identified as MMP-8, also known as collagenase 2 [13]. The severity of periodontal disease is directly correlated with the level of MMPs that can be found in both saliva and GCF [14,15]. Thus, for the purpose of monitoring periodontal and peri-implant diseases, several studies have investigated the diagnostic potential of MMPs and tissue inhibitors of metalloproteinases (TIMPs) in oral fluids as chair-side or point-of-care test markers [16,17]. Additionally, a new study showed that MMPs present in GCF from teeth with AAP have the ability to be used as markers for diagnosis [18].

The last cells to influence bone resorption in AP are osteoclasts, which make them an indicator of the healing and progression stages of the AP. The differentiation and activation of osteoclasts are partly regulated by the interaction of the receptor activator of nuclear factor κB (RANK), its ligand (RANKL), and its decoy receptor called osteoprotegerin (OPG). Thus, RANKL levels in AP have been reported to be significantly higher than in healthy tissues [19]. On the other hand, the association between these biomarkers such as the RANKL/OPG ratio has been shown to be a reliable marker of AP progression [20,21,22].

Despite the recent advances in the imaging process, the molecular changes associated with SAP and AAP are yet to be examined, and the bone resorptive dynamics in symptomatic and asymptomatic states of apical periodontitis remain unknown [1,2]. Moreover, the biomarkers in GCF might help to indicate the presence of a disease process before extensive clinical damage has occurred. Therefore, this study aims to investigate the abilities of GCF biomarker levels of MMP8, TIMP1, RANK, RANKL, and OPG to differentiate between SAP and AAP in patients with AP.

## 2. Materials and Methods

### 2.1. Study Design

**Figure 1:**
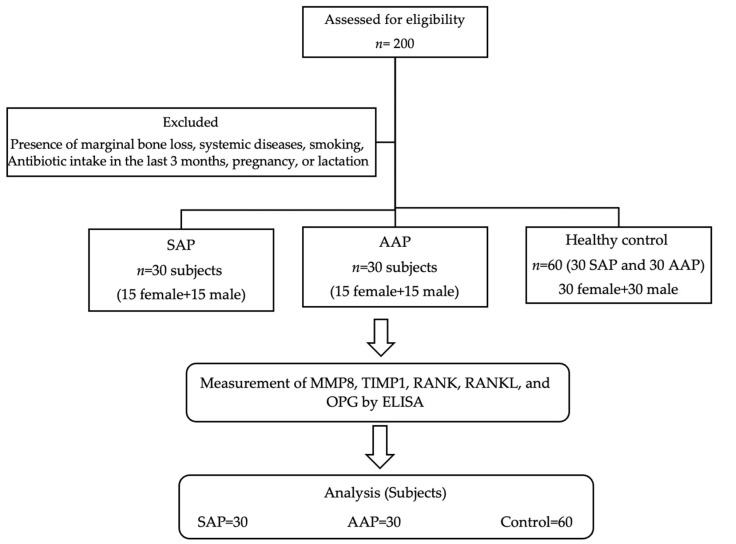
Flow diagram of the study.

This was a cross-sectional case–control study in which teeth with AP (SAP and AAP) were considered cases, and healthy teeth were used as controls (Figure 1).

Participants were selected from patients referred to the Department of Operative Dentistry and Endodontics, College of Dentistry, University of Baghdad, for endodontic treatment from May 2022 to May 2023. The Ethics Committee of the College of Dentistry, University of Baghdad approved the study protocol (Ref.# 546 on 17 April 2022) in accordance with the Helsinki Declaration for human research. Patient information sheets containing the study’s nature and aim were provided to all patients before signing the consent form.

Exclusion criteria for all subjects included the presence of marginal bone loss, gingivitis, systemic diseases and disorders, pregnancy, smoking, and the intake of medication that can influence periodontal tissue turnover in the 3 months prior to the beginning of the study.

### 2.2. Study Participants

Sixty patients (30 with SAP and 30 with AAP) were recruited. The examined teeth were maxillary first or second premolar teeth with either SAP or AAP. A healthy collateral tooth was selected as a control. Following the collection of the demographic data, GCF samples were collected from the selected teeth after clinical and radiographic examinations.

A preoperative cone beam computed tomography (CBCT) with a limited field of view (FOV) was programmed with as low as reasonably achievable principles in accordance with the joint position statement for the use of CBCT in endodontics by the American Association of Endodontists and the American Academy of Oral and Maxillofacial Radiology [23]. All CBCTs were evaluated to identify the bone lesion within selected cases to be limited in the periapical cancellous bone; therefore, any lesions that extended to the cortical bone were excluded.

**Figure 2:**
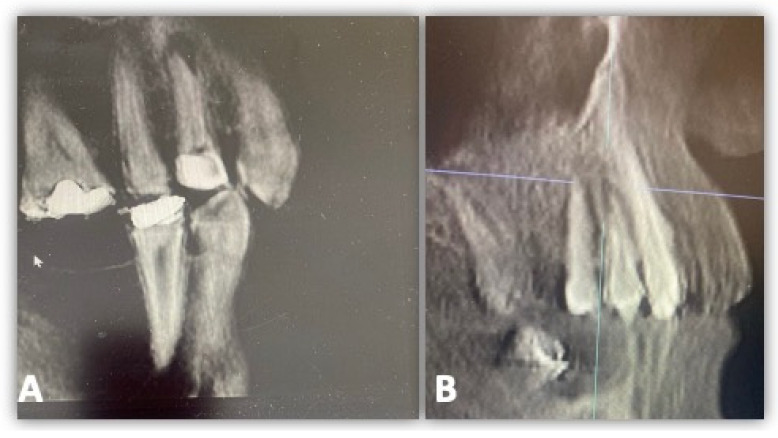
Radiographical CBCT images of cases included in this study: (**A**) tooth #5 with AAP, (**B**) another case tooth #5 with SAP.

The AP teeth were diagnosed clinically by the presence or absence of symptoms for SAP and AAP. The SAP symptoms were pain and tenderness to percussion, while the AAP symptoms were no pain and tenderness. Both SAP and AAP showed periapical lesions of different sizes with no peripheral bone destruction, as seen in Figure 2A,B.

### 2.3. Study Criteria

The recruited participants had AP in one or both maxillary premolars on one side and the same clinically healthy on the other side. All recruited participants had clinically healthy gingiva with normal marginal gingiva and periodontium with no evidence of bone destruction and no bleeding on probing. Exclusion criteria for all subjects included the presence of perio-endo lesions, marginal bone loss, systemic diseases and disorders, pregnancy, smoking, and medication intake that could have influenced periodontal tissue in the last three months.

### 2.4. GCF Samples Collection and Analysis

**Figure 3:**
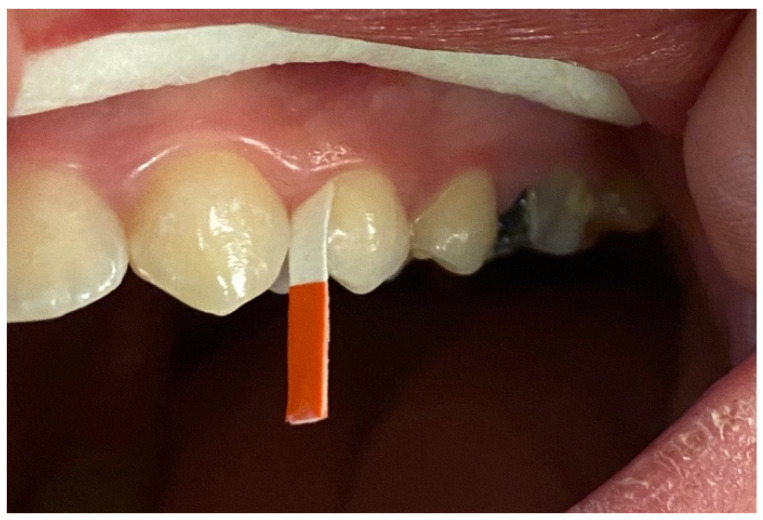
Intra-crevicular collection of GCF.

GCF samples were collected at the mesial, distal, and lingual surfaces of the selected teeth by Periopaper strips (Oraflow Inc., New York, NY, USA) using the intra-crevicular method (Figure 3).

The site of the tooth was isolated with a cotton roll, and supragingival plaque was removed manually using a gentle wiping movement with a clean gauze [24]. Tweezers were used to gently insert Periopaper strips into the depth of the gingival sulcus until minimal resistance was felt. Each paper was left on site for 30 s before removal and direct inspection for any sign of contamination with saliva or blood. The samples were transferred to a pre-weighed Eppendorf tube containing 300 μL of phosphate-buffered saline (PBS) and stored at −80 °C until analysis. The GCF samples per tooth were pooled as representative of the selected tooth. The volume of the collected GCF was determined as previously described [25,26]. Briefly, the GCF was determined using the formula below:

W_GCF_ = W_2_ − W_1_,

W_GCF_ = GCF (μg), W_2_ = Eppendorf tube wight with PBS (300 μL) and Periopaper following GCF collection, and finally, W_1_ = pre-weighed Eppendorf tube with PBS (300 μL). The weight of the Periopaper was deducted from the weight of W_GCF_ to determine the weight of the GCF. Lastly, assuming that the GCF had a density of 1 mg/mL, the volume was changed from μg to μL. This was carried out with the following formula [27].

volume = Mass/Density’.

All samples were stored in a −80 °C freezer, a critical step to preserve the integrity of the samples, until the time of testing.

The samples were thawed and then centrifuged at 400–500× *g* for 4–5 min, and the concentrations were estimated using ELISA kits, according the manufacturer’s instructions for MMP8 (Shanghai YL Biotech Co., Ltd., Shanghai, China), TIMP1 (Shanghai YL Biotech Co., Ltd., Shanghai, China), RANK (Shanghai YL Biotech Co., Ltd., Shanghai, China), RANKL (Shanghai YL Biotech Co., Ltd., Shanghai, China), and OPG (Shanghai YL Biotech Co., Ltd., Shanghai, China). The optical density was measured at 450 nm using a microplate reader (GloMax^®^, Promega, Madison, WI, USA) and then the optical density was then converted into a corresponding concentration (ng/mL) for each cytokine using a specific equation obtained from a standard curve being plotted by an independent investigator not involved in the sample collection to ensure the blindness of the study. This obtained value was the concentration (in 1 mL) of the biomarkers eluted in 300 μL; thus, the final concentration of each biomarker in the original GCF volume was calculated using the formula below [28]:

ELISA output × 0.3/GCF volume (μL).

### 2.5. Pilot Study and Sample Size Calculation

A pilot study was initially conducted on 5 patients not included in the analysis. In total, fifteen samples were analyzed by ELISA, and the level of the MMP8 biomarker was assigned to calculate the sample size using the formula below [29]:

Sample size = r + 1/r × (SD) 2 × (Zβ + Zα/2)2/d2

In the above example, d is the predicted mean difference between cases and controls, Zβ is the standard normal variation with a power of 90%, which is 1.28, Zα/2 is a 5% type 1 error, which is 1.96, and r is the ratio of cases to controls, which is 2. In order to compensate for attrition from the sample, the estimated sample size for the periodontitis group (which was 26), was rounded to 30. Consequently, all study groups comprised 15 male and 15 female patients.

### 2.6. Statistical Analysis

Data were assessed for normality using a Shapiro–Wilk test. A Mann–Whitney U test was used to compare the biomarker levels between groups. Spearman’s correlation was used to examine the correlation between the study biomarkers. The accuracy of the examined biomarkers was examined using a receiver operating characteristic (ROC) curve and the area under the curve (AUC) to differentiate between SAP, AAP, and the control sites. Threshold points for biomarker levels were selected from the ROCs with the highest sensitivity and specificity. The level of significance was set at *p* < 0.03 for multigroup comparisons. All calculations were performed using the SPSS software package (version 26; SPSS Inc., Chicago, IL, USA).

## 3. Results

A total of 200 patients were assessed for eligibility. The final analysis included 60 patients, equally distributed among the two study groups. The demographic variables of the participants are summarized in Table 1.

**Figure 4:**
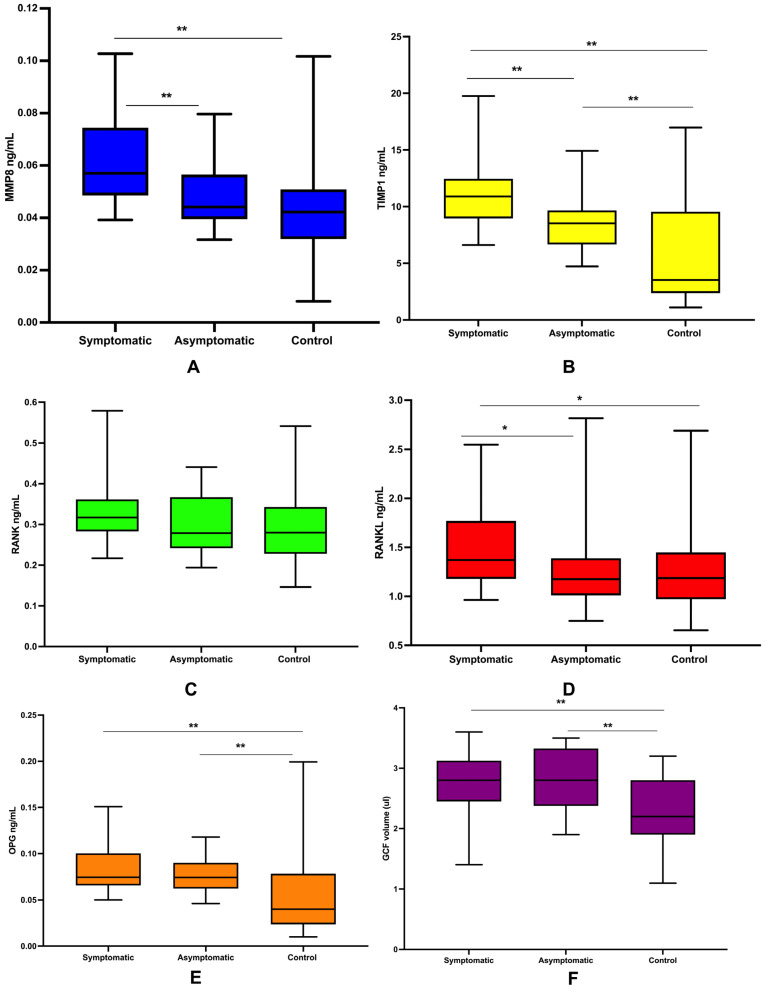
Shows the comparison of biomarkers level among all examined sites: (**A**) the level of MMP8 ng/mL; (**B**) the level of TIMP1 ng/mL; (**C**) the level of RANK ng/mL; (**D**) the level of RANKL ng/mL; (**E**) the level of OPG ng/mL; and (**F**) the level of GCF volume; * *p* < 0.05; ** *p* < 0.01.

The median levels of MMP8 in SAP were statistically significantly higher than in AAP and the control group (Figure 4A). Further, the median levels of TIMP1 were statistically significantly different across all study groups (Figure 4B). However, these differences were not detected for RANK (Figure 4C). Regarding RANKL, the median level was statistically significantly higher in SAP compared to AAP and the control teeth (Figure 4D). Finally, the median levels of OPG and GCF volumes in both SAP and AAP were statistically significantly higher than at the control site (Figure 4E,F).

Correlations between biomarker levels were examined in both SAP and AAP sites by Spearman’s correlation (Table 2). Statistically significant correlations were identified between all examined biomarkers in SAP sites, as the strongest correlations were found between TIMP1 and OPG (0.83), RANK and RANKL (0.82), MMP8 and RANKL (0.82), and MMP8 and RANK (0.79). Similarly, in AAP sites, statistically significant associations were detected between all examined biomarkers apart from TIMP1 and RANK (*p* > 0.05).

**Figure 5:**
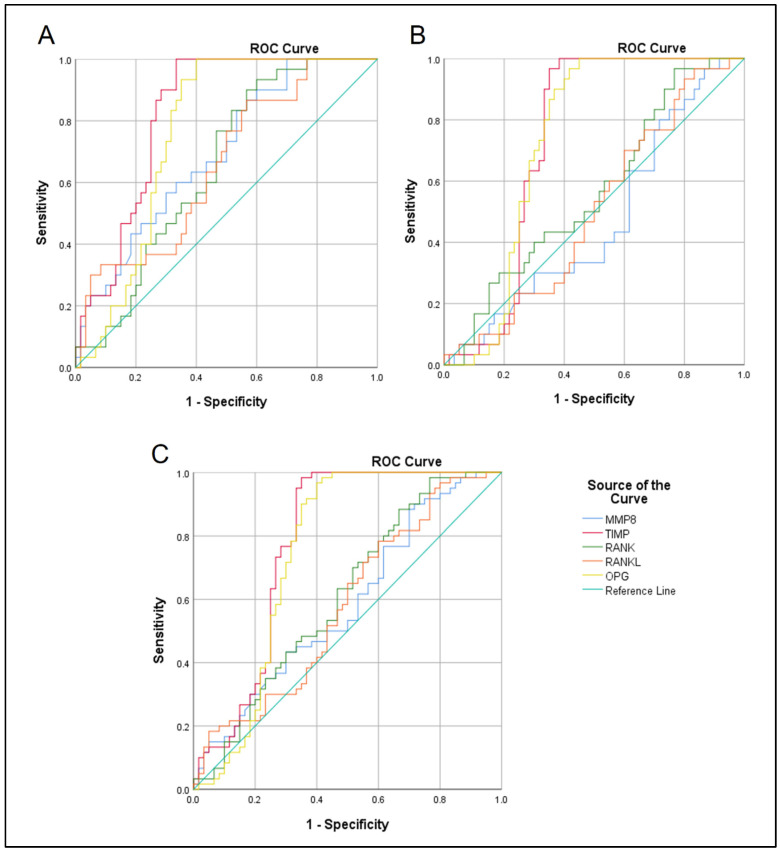
ROC curves for examined GCF biomarkers to differentiate between SAP and the control group (**A**), AAP and the control group (**B**), and SAP and AAP (**C**).

ROC curves were used to evaluate the capability of the examined biomarkers to differentiate between SAP and the control group (Figure 5A), AAP and the control group (Figure 5B), and finally between SAP and AAP (Figure 5C). Statistically significant differences were apparent in the ability of all tested biomarkers to differentiate between SAP and the controls (*p* < 0.05). Additionally, TIMP1 and OPG showed the highest abilities with an AUC of 0.824 (CI: 0.74–0.9) and 0.763 (CI: 0.66–0.86), respectively, followed by MMP8 (AUS: 0.694, CI: 0.58–0.8), RANKL (AUS: 0.656, CI: 0.53–0.77), and RANK (AUS: 0.652, CI: 0.54–0.76) (Figure 5A and Table 3). The study results also identified a statistically significant difference (*p* < 0.05) between AAP and the control sites, but only with TIMP1 (AUC: 0.732, CI: 0.62–0.83) and OPG (AUC: 0.73, CI: 0.62–0.83) (Figure 5B). Importantly, only TIMP1, OPG, and RANK revealed statistically significant differences in distinguishing between SAP and AAP with an AUS of 0.778 (CI: 0.68–0.86), 0.747 (CI: 0.64–0.84), and 0.607 (CI: 0.51–0.71), respectively (Figure 5C and Table 3). These findings have direct implications for clinical practice, suggesting the potential use of TIMP1 and OPG in differentiating between these conditions.

## 4. Discussion

The key finding of the current study was that TIMP1 and OPG showed the highest abilities in differentiating between SAP and AAP. Moreover, MMP8, RANK, and RANKL could only distinguish between SAP and the control groups. These patterns were not detected when compared between AAP vs. the control group and SAP vs. AAP. Therefore, TIMP1 and OPG exhibited the potential to discriminate between different types of AP and healthy teeth. Indeed, finding biomarkers that can aid in distinguishing different types of AP is of great value to clinical practice. The rationale behind the current study was based on these premises and aimed to examine the biomarkers’ capabilities to differentiate SAP, AAP, and controls, which, to the best of our knowledge, has yet to be explored. 

This study examined the inflammatory biomarkers within GCF, which were challenging to collect and had some practical limitations compared to saliva. However, GCF reveals the site-specific nature of the disease and cannot be influenced by salivary flow [30]. Indeed, salivary samples were not suitable for the current study as all studied teeth belonged to the same patient. On the other hand, the future holds promising possibilities for GCF as a tool that offers a non-invasive, efficient, and easy-to-use approach to sample the biomarkers of inflammation and bone resorption in dental and periodontal disease [17].

MMP8 showed higher levels in the SAP group compared to the AAP and control groups. This can be attributed to the fact that during AP, MMP8 is significantly released by neutrophils and plays a significant role in periodontal tissue destruction, particularly in the degradation of type-I collagen, which is the main constituent of the periodontal extracellular matrix [15,31]. Similarly, TIMP1 levels showed significantly higher concentrations in SAP than in both AAP and control groups. As degradation of the periodontal connective tissues is a critical component of periodontitis, TIMP1 has therapeutic value in regulating the activity of MMP enzymes involved in this pathogenic process. Indeed, an imbalance in the regulation of MMP activity can lead to tissue destruction, fibrosis, and degradation of the ECM, representing various stages of disease progression [32]. Thus, the low level of TIMP1 found in the present study is understandable as the AP is absent, which is commensurate with the results of a previous study [33].

Other longitudinal studies [34,35] demonstrated decreased TIMP-1 levels within active sites of progressive periodontitis and provided evidence that tissue destruction results from an imbalance of MMPs over their tissue inhibitor (TIMP1). Although in the present study, the levels of MMP8 were comparable between AAP and controls, the TIMP1 level was still significantly higher within AAP samples than in the control. GCF biomarkers may be elevated in the active phase of the disease when the lesion starts to develop, and this could be related to the dynamics of AAP, which are characterized by minor lesion expansion and limited bone resorption [36]. In order to verify the effectiveness of the intervention and detect the onset of periapical lesions, the suggested approach may thus be applied in the short-term follow-up of endodontically treated teeth with healed periodontal issues.

The function of osteoclasts mainly involves bone resorption regulated by OPG which binds to RANK-L, preventing interaction with RANK; thus, differentiation of osteoclasts will be inhibited. It is important to note that the mechanism of osteoclastogenesis and alveolar bone resorption depends on the interaction between these molecules. Although these three markers were detected within SAP and AAP, only OPG in the SAP group showed statistically significantly higher levels compared to the AAP and the control groups. A possible explanation could be that only symptomatic lesions might represent an active stage of AP, as reflected by concomitantly elevated levels of OPG and RANKL [37]. Furthermore, activation pathways other than RANK might be induced in AAP, resulting in a non-significant difference in the RANK level in the study groups [38]. Finally, the lower level of RANKL OPG in the control group might represent normal bone homeostasis [39].

Interestingly, positive correlations were identified between all examined biomarkers in both SAP and AAP groups except between TIMP1 and RANK in the AAP group. This can be explained by the fact that these biomarker levels are highly expressed during inflammation, and their mechanisms of action are strongly correlated. For example, TIMP1 is a natural inhibitor of MMP8, and its levels are expected to be strongly correlated [40]. Similarly, RANK, RANKL, and OPG all together play a significant role in the differentiation and activation of osteoclasts in periodontitis [41], and their expression levels would be expected to be highly correlated.

The investigated biomarkers in the present study have already shown a high diagnostic capability for periodontal disease. However, amongst the examined biomarkers in this study, only TIMP1 and OPG consistently showed the highest capabilities in distinguishing between SAP, AAP, and the control groups. This can be associated with a difference in the nature of periodontal disease, where the disease happens at the margin of the gingiva, whereas in AP, the condition is more profound at the apical region of the tooth. Indeed, the potential capability of the biomarkers has been shown to differ between these two diseases [42]. To the best of our knowledge, this is the first study to show the abilities of TIMP1 and OPG in GCF for AP in distinguishing between SAP, AAP, and healthy sites.

This study has some limitations, including the time required to collect and analyze the GCF samples, which could limit the applicability of this approach in daily clinical practice. Furthermore, as this study is observational, only the association can be examined, while the causality of these biomarkers for AP has yet to be explored in clinical trials. Nevertheless, the current study is one of the few studies that explore the potential of GCF biomarkers to discriminate between SAP, AAP, and healthy teeth. Additionally, TIMP1 and OPG showed promising capabilities in distinguishing between SAP, AAP, and healthy sites, which can be explored in future studies.

## 5. Conclusions

This study revealed that GCF biomarker levels of TIMP1 and OPG exhibit high capabilities in discriminating between SAP, AAP, and healthy teeth. These findings represented a further step towards developing a personalized AP treatment through identifying AP disease status for more optimized and accurate treatment planning and prognosis. Other possible biomarkers with higher diagnostic potential need to be examined, and clinical trial studies are required to verify the results of the current study.

## Figures and Tables

**Table 1:** Demographic data of the patients.

Variable		Mean ± SD	Number (*n*)	Percent (%)
**Sex**	Male		30	50
	Female		30	50
**Age Groups (year)**	20–29	26.47 ± 2.096	15	25
	30–39	35.28 ± 2.598	23	38
	40–49	43.27 ± 3.006	22	37

SD, Standard deviation.

**Table 2:** Correlation between all biomarkers in SAP and AAP sites.

		SAP					AAP				
		MMP8	TEMP1	RANK	RANKL	OPG	MMP8	TEMP1	RANK	RANKL	OPG
**MMP8**	**R**		0.48 **	0.79 **	0.82 **	0.45 **		0.22 *	0.74 **	0.79 **	0.28 **
	***p* value**		0.0001	0.0001	0.0001	0.0001		0.037	0.0001	0.0001	0.007
**TEMP1**	**R**			0.38 **	0.48 **	0.83 **			0.202	0.26 *	0.84 **
	***p* value**			0.0001	0.0001	0.0001			0.58	0.013	0.0001
**RANK**	**R**				0.82 **	0.46 **				0.81 **	0.3 **
	***p* value**				0.0001	0.0001				0.0001	0.005
**RANKL**	**R**					0.49 **					0.33 **
	***p* value**					0.0001					0.002

R is the correlation coefficient, * Correlation is significant at the 0.05 level, ** Correlation is significant at the 0.01 level.

**Table 3:** Properties of specific thresholds of tested biomarkers to differentiate between SAP and the control group, AAP and the control group, and SAP and AAP.

Groups Comparison	Variables	Threshold µg/mL	Sensitivity%/Specificity%	AUC	95% CI	*p* Value
**SAP vs. control**	MMP8	0.053	60/67	0.694	0.58–0.8	0.003
	TIMP1	8.64	80/75	0.824	0.74–0.9	0.0001
	RANK	0.31	60/57	0.652	0.54–0.76	0.019
	RANKL	1.27	57/57	0.656	0.53–0.77	0.017
	OPG	0.066	73/70	0.763	0.66–0.86	0.0001
**AAP vs. control**	MMP8	0.046	43/44	0.47	0.34–0.59	0.647
	TIMP1	7.46	66/67	0.732	0.62–0.83	0.0001
	RANK	0.26	56/50	0.562	0.44–0.68	0.338
	RANKL	1.16	53/49	0.502	0.38–0.62	0.973
	OPG	0.067	70/70	0.73	0.62–0.83	0.0001
**SAP vs. AAP**	MMP8	0.049	53/50	0.582	0.48–0.68	0.122
	TIMP1	7.78	75/72	0.778	0.68–0.86	0.0001
	RANK	0.29	53/54	0.607	0.51–0.71	0.043
	RANKL	1.21	56/54	0.579	0.47–0.68	0.136
	OPG	0.067	70/70	0.747	0.64–0.84	0.0001

## Data Availability

The data are available from the corresponding author upon reasonable request.
